# Enzyme replacement therapy and immunotherapy lead to significant functional improvement in two children with Pompe disease: a case report

**DOI:** 10.1186/s13256-024-04638-5

**Published:** 2024-07-18

**Authors:** Sandra Milena Castellar-Leones, Fernando Ortiz-Corredor, Daniel Manrique-Hernández, Diana Sánchez-Peñarete, Edicson Ruiz-Ospina, Diana Soto-Peña, Cristian Correa-Arrieta

**Affiliations:** 1 Facultad de Medicina, Universidad Nacional de Colombia, Carrera 30 No. 45-03. Edificio 471, Piso 5to, Of. 513-A, Bogotá, Colombia; 2 Centro de Investigación en Fisiatría y Electrodiagnóstico, CIFEL, Bogotá, Colombia; 3 Hospital Universitario Nacional de Colombia, Bogotá, Colombia; 4 Instituto Roosevelt, Bogotá, Colombia; 5 Universidad de la Sabana, Chía, Colombia; 6 Hospital Universitario San Ignacio, Bogotá, Colombia

**Keywords:** Pompe disease, Enzyme replacement therapy (ERT), Acid alpha-glucosidase (GAA), Lysosomal storage disorders

## Abstract

**Background:**

Pompe disease, a rare autosomal recessive disorder caused by acid alpha-glucosidase deficiency, results in progressive glycogen accumulation and multisystem dysfunction. Enzyme replacement therapy with recombinant human acid alpha-glucosidase is the standard of care; however, some patients develop anti-recombinant human acid alpha-glucosidase antibodies, leading to reduced efficacy. This case report presents two infants with early-onset Pompe disease who developed IgG antibodies to enzyme replacement therapy and were subsequently treated with methotrexate, highlighting the importance of monitoring antibody development and exploring alternative therapeutic approaches.

**Case presentation:**

Patient 1, a 10-month-old female from Bogota, Colombia, presented with generalized hypotonia, macroglossia, hyporeflexia, and mild left ventricular hypertrophy. Diagnostic tests confirmed early-onset Pompe disease, and enzyme replacement therapy was started at 12 months. Due to a lack of improvement and high anti-recombinant human acid alpha-glucosidase IgG antibody titers (1:1800), methotrexate was started at 18 months. After 8 months of combined therapy, antibody titers were negative and significant improvement in motor function was observed using the Gross Motor Function Measure 88. Patient 2, a 7-year-old female from Bogota, Colombia, was diagnosed with early-onset Pompe disease at 12 months and initiated enzyme replacement therapy. At 5 years of age, she experienced frequent falls and grip strength alterations. Functional tests revealed motor development delay, generalized hypotonia, and positive anti-recombinant human acid alpha-glucosidase IgG antibody titers (6400). Methotrexate was initiated, leading to a reduction in falls and antibody titers (3200) after 6 months, with no adverse events or complications. Motor function improvement was assessed using the Motor Function Measurement 32.

**Conclusions:**

The presented cases highlight the importance of monitoring patients for anti-recombinant human acid alpha-glucosidase antibody development during enzyme replacement therapy and the potential benefit of methotrexate as an immunomodulatory agent in early-onset Pompe disease. Early diagnosis and timely initiation of enzyme replacement therapy, combined with prophylactic immune tolerance induction, may improve clinical outcomes and reduce the development of anti-recombinant human acid alpha-glucosidase antibodies. The cases also highlight the importance of objective motor function assessment tools, such as Gross Motor Function Measure 88 and Motor Function Measurement 32, in assessing treatment response. Further research is needed to optimize treatment regimens, monitor long-term effects, and address the current limitations of enzyme replacement therapy in Pompe disease.

## Introduction

Pompe disease, also known as type II glycogenosis or acid maltase deficiency, is a rare autosomal recessive disorder first described by the Dutch pathologist Johannes Pompe in 1932. The incidence of Pompe disease varies between 1/40,000 and 1/400,000 depending on the population and geographical region studied [1, 2]. The disease is caused by a deficiency in the enzyme acid alpha-glucosidase (GAA), which is encoded by the *GAA* gene located on chromosome 17q25.2-q25.3 [3]. Deficiency of GAA results in progressive accumulation of lysosomal glycogen, leading to cellular dysfunction and tissue damage.

Pompe disease is a multisystem disorder that can affect individuals of any age and presents with a variety of signs and symptoms, including progressive muscle weakness, hypotonia, delayed motor development, gait disturbance, respiratory distress, cardiomyopathy, and macroglossia. The onset, severity, and progression of symptoms can vary widely between individuals, depending on the age of onset, the extent of the disease, and the level of residual GAA activity in the tissues [3].

Diagnosis of Pompe disease typically involves a thorough evaluation of clinical features, including a complete physical examination and detailed medical history, as well as laboratory and genetic testing. Enzyme assays in isolated lymphocytes or fibroblast cultures can confirm the diagnosis by demonstrating a deficiency in acid alpha-glucosidase activity. In addition, molecular studies of the *GAA* gene can identify specific mutations associated with the disease. Accurate and timely diagnosis of Pompe disease is essential for effective management of the disease. With early detection, people with Pompe disease can benefit from treatment strategies such as enzyme replacement therapy, which can improve symptoms, slow disease progression, and prolong survival [3].

Enzyme replacement therapy (ERT) is the only approved treatment for Pompe disease and has been available since the Food and Drug Administration (FDA) approved recombinant human acid alpha-glucosidase (rhGAA; alglucosidase alfa) in 2006. Administered through biweekly infusions of recombinant human GAA, ERT has been shown to reduce complications and preserve motor, respiratory, and cardiac function in patients with Pompe disease, especially when started early. Despite the benefits of ERT, some patients may develop antibodies to the therapy, leading to reduced efficacy [4]. Therefore, monitoring patients for antibody development and identifying appropriate treatment strategies is critical.

In this context, we present a case report of two infants with early-onset Pompe disease (EOPD) who initially failed to respond to ERT and subsequently developed IgG antibodies to the therapy. The patients were treated with methotrexate, and their functional outcome was objectively assessed by motor function testing. This case report highlights the importance of monitoring patients for the development of antibodies to ERT and the need for alternative therapeutic approaches when ERT is no longer effective.

## Case presentations

### Patient 1

A 10-month-old female infant from Bogota, Colombia, was suspected of having early-onset Pompe disease (EOPD) on the basis of her family history and clinical presentation. The patient was born by cesarean section at 39 weeks without complications, weighing 3790 g and measuring 47 cm in length. A history of threatened abortion at 12 weeks’ gestation was reported. The patient’s brother had a history of hypotonia, delayed motor development, and hypertrophic cardiomyopathy resulting in his death at 15 months of age.

Physical examination revealed generalized hypotonia, macroglossia, and hyporeflexia. A transthoracic echocardiogram showed mild hypertrophy of the left ventricular myocardium. The dry blood spot test showed abnormal results with a neutral/inhibited alpha-glucosidase ratio of 43.7 (normal values < 16) and an alpha-glucosidase inhibition percentage of 89% (normal values < 86%). Alpha-glucosidase activity was low at 0.14 nmol/mL (normal range 1.29–25.7 nmol/L). Sequencing of the *GAA* gene identified two pathogenic variants: c.1064 T > C (p.Leu355Pro, exon 6, heterozygous) and c.1465G > A (p.Asp489Asn, heterozygous). Laboratory tests showed elevated levels of AST, ALT, and CPK. Functional motor assessment revealed delayed motor development and generalized hypotonia.

At the age of 12 months, the patient was started on enzyme replacement therapy (ERT) with recombinant human alpha-glucosidase (rhGAA; alglucosidase alfa) at a dose of 20 mg/kg every 2 weeks. However, no improvement in motor function was observed after 18 months. Quantification of anti-rhGAA IgG antibody titers showed a result of 1:1800 (reference range 0–100). Therapy with methotrexate (MTX) was started at a dose of 15 mg/m^2^ every 2 weeks.

**Fig. 1:**
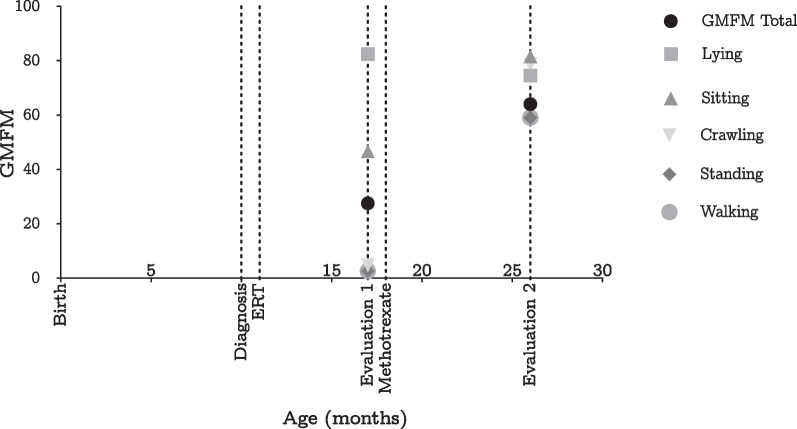
Changes in Gross Motor Function Measure 88 (GMFM-88) after initiation of immunomodulation. Source: authors’ elaboration

At the age of 26 months, after 8 months of MTX and continued ERT, a follow-up anti-rhGAA IgG antibody titer test was reported as negative. Motor function assessment showed significant improvement, with the patient able to walk more than ten steps independently, turn around, walk at an accelerated pace, and transition from standing to sitting with support (Fig. 1). No adverse events or respiratory or cardiac complications were observed, and the patient maintained a good nutritional status.

### Patient 2

The second case is a 7-year-old female patient from Bogota, Colombia, who was diagnosed with EOPD at the age of 12 months. The diagnosis was confirmed by a dry blood spot test that showed a neutral/inhibited alpha-glucosidase ratio of 37.4 (normal values < 16) and an alpha-glucosidase inhibition percentage of 90% (normal values < 86%). GAA enzyme activity in leukocytes also showed a neutral/inhibited alpha-glucosidase ratio and alpha-glucosidase inhibition percentage above the reference range. Sequencing of the *GAA* gene identified two pathogenic variants: c.1064T > C (p.Leu355Pro, exon 6, heterozygous) and c.2560C > T (p.Arg854Ter, exon 18, heterozygous).

**Fig. 2:**
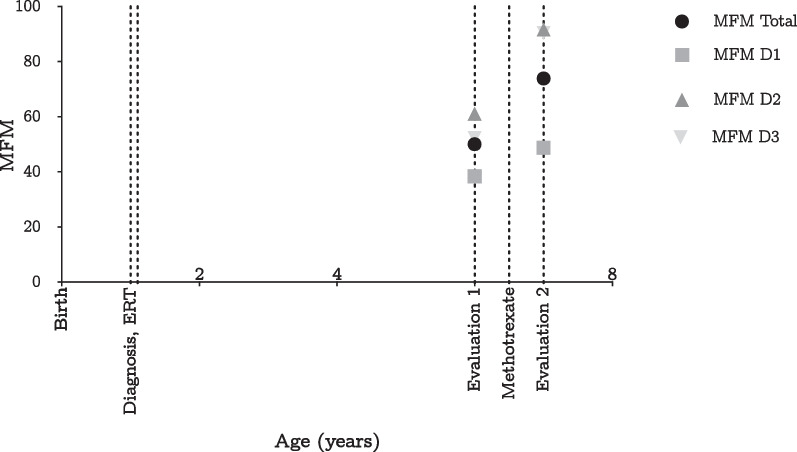
Changes in Motor Function Measurement 32 (MFM) after initiation of immunomodulation. Source: Authors' elaboration

ERT with recombinant human alpha-glucosidase (rhGAA; alglucosidase alfa) at a dose of 20 mg/kg every 2 weeks was started at 12 months of age. Although the patient met neurodevelopmental milestones, she began to experience frequent falls and changes in grip strength at 5 years of age. At 6 years of age, functional testing revealed a 6-minute walk distance of 260 m, a speed of 0.72 m/second, bilateral foot drop, and hip flexion deformities. Functional motor assessment revealed delayed motor development and generalized hypotonia (Fig. 2).

The frequency of falls and grip changes increased with age. Quantification of the anti-rhGAA IgG antibody titer showed a positive value of 6400. Consequently, treatment with MTX at a dose of 15 mg/m^2^ every 2 weeks was initiated, resulting in a reduction in falls. After 6 months, a follow-up functional assessment and quantification of anti-rhGAA IgG antibody titers was performed, showing a decrease in anti-rhGAA IgG antibody titers to 3200 from the previous measurement. The patient responded well to ERT with no adverse events or respiratory or cardiac complications, while maintaining good nutritional status and independent ambulation.

## Discussion and conclusions

Pompe disease, also known as type II glycogenosis, is a lysosomal storage disorder with a clinical spectrum ranging from the classic infantile form, which is the most severe, to late-onset forms. The advent of enzyme replacement therapy (ERT) with alglucosidase alfa (rhGAA) has dramatically changed the natural history of this disease, significantly improving survival and clinical outcomes [5]. However, the development of anti-rhGAA antibodies remains a major challenge that can compromise both the safety and efficacy of ERT [6].

The cases presented so far illustrate the benefits of ERT and prophylactic immune tolerance induction (ITI) in patients with classical infantile Pompe disease. The results of these cases are consistent with literature reports showing that ERT combined with ITI can improve clinical outcomes and reduce the development of anti-rhGAA antibodies in patients with negative cross-reactive immunological material (CRIM) status [5, 7, 8].

Early diagnosis and timely initiation of ERT are critical to achieving optimal motor outcomes in children with Pompe disease. The earlier that treatment is initiated, the greater the likelihood of preventing or reversing irreversible muscle damage [8]. In the cases presented, patients who received ERT and ITI at a younger age, such as patient 1 (3.8 months) and patient 2 (3.0 months), achieved motor milestones such as independent walking, supporting current recommendations to initiate ERT as early as possible [9, 10].

The impact of ERT on motor, respiratory, and cardiac function in patients with Pompe disease has been well documented in the literature [11]. In the cases presented, significant improvements in motor function were observed, with patients achieving independent ambulation and age-appropriate developmental milestones. In addition, significant reductions in left ventricular mass index (LVMI) have been observed, indicating an improvement in cardiac hypertrophy [8, 9]. These findings support the benefits of ERT on cardiac and motor function in patients with Pompe disease.

The use of methotrexate to control the development of anti-rhGAA antibodies in patients with Pompe disease treated with ERT has been evaluated in preclinical and clinical studies [5]. In the cases presented, methotrexate was used as part of a prophylactic ITI regimen. This strategy was effective in inducing immune tolerance to ESRD in most patients, with 88% of patients maintaining low or negative anti-rhGAA antibody titers [10]. These findings are consistent with literature reports suggesting that methotrexate may be an effective alternative to rituximab in preventing the development of anti-rhGAA antibodies [5, 12].

Both the presented clinical cases and the reviewed articles have strengths and limitations that should be considered. A major strength is the cohort size and long-term follow-up in some cases, which provides valuable information on the safety and efficacy of ERT and prophylactic ITI [8, 10]. However, a common limitation is the retrospective design of some studies and the lack of an adequate control group in others. In addition, the heterogeneity of treatment regimens and clinical endpoints evaluated makes direct comparisons between studies difficult.

In conclusion, the cases presented and the existing literature highlight the importance of early diagnosis and timely initiation of ERT in patients with classic infantile Pompe disease. ERT in combination with prophylactic ITI with methotrexate has been shown to be effective in improving clinical outcomes, including motor, respiratory, and cardiac function, and in reducing the development of anti-rhGAA antibodies. However, further research is needed to optimize treatment regimens, monitor long-term effects, and address current limitations of ERT, such as the inability to cross the blood–brain barrier and variability in skeletal muscle response. Future prospective studies and controlled clinical trials will be essential to further improve the management and outcomes of patients with Pompe disease.

## Data Availability

The data sets analyzed in the current study are available from the corresponding author on reasonable request.

## Abbreviations

ALT: Alanine transaminase
AST: Aspartate transaminase
CPK: Creatine phosphokinase
CRIM: Cross-reactive immunological material
EOPD: Early-onset Pompe disease
ERT: Enzyme replacement therapy
GAA: Acid alpha-glucosidase
GMFM: Gross Motor Function Measure
IgG: Immunoglobulin G
ITI: Immune tolerance induction
LVMI: Left ventricular mass index
MFM: Motor function measurement
MTX: Methotrexate
rhGAA: Recombinant human acid alpha-glucosidase
